# Single-Cell Gene Profiling Reveals Social Status-Dependent Modulation of Nuclear Hormone Receptors in GnRH Neurons in a Male Cichlid Fish

**DOI:** 10.3390/ijms21082724

**Published:** 2020-04-15

**Authors:** Satoshi Ogawa, Ishwar S. Parhar

**Affiliations:** Brain Research Institute, Jeffrey Cheah School of Medicine and Health Sciences, Monash University Malaysia, Bandar Sunway, Subang Jaya 47500, Selangor, Malaysia; ishwar@monash.edu

**Keywords:** steroids, xenobiotic, aggression, social stress, teleosts

## Abstract

Gonadotropin-releasing hormone (GnRH) is essential for the initiation and maintenance of reproductive functions in vertebrates. To date, three distinct paralogue lineages, GnRH1, GnRH2, and GnRH3, have been identified with different functions and regulatory mechanisms. Among them, hypothalamic GnRH1 neurons are classically known as the hypophysiotropic form that is regulated by estrogen feedback. However, the mechanism of action underlying the estrogen-dependent regulation of GnRH1 has been debated, mainly due to the coexpression of low levels of estrogen receptor (ER) genes. In addition, the role of sex steroids in the modulation of GnRH2 and GnRH3 neurons has not been fully elucidated. Using single-cell real-time PCR, we revealed the expression of genes for estrogen, androgen, glucocorticoid, thyroid, and xenobiotic receptors in GnRH1, GnRH2, and GnRH3 neurons in the male Nile tilapia *Oreochromis niloticus*. We further quantified expression levels of estrogen receptor genes (ERα, ERβ, and ERγ) in three GnRH neuron types in male tilapia of two different social statuses (dominant and subordinate) at the single cell level. In dominant males, GnRH1 mRNA levels were positively proportional to ERγ mRNA levels, while in subordinate males, GnRH2 mRNA levels were positively proportional to ERβ mRNA levels. These results indicate that variations in the expression of nuclear receptors (and possibly steroid sensitivities) among individual GnRH cells may facilitate different physiological processes, such as the promotion of reproductive activities through GnRH1 neurons, and the inhibition of feeding and sexual behaviors through GnRH2 neurons.

## 1. Introduction

Gonadotropin-releasing hormone (GnRH, also known as luteinizing hormone-releasing hormone, LHRH) is essential for the initiation and maintenance of reproductive functions. Over forty known structural variants of GnRH types have been identified in vertebrates and invertebrates [1], which are classified into three distinct paralogues lineages, i.e., GnRH1, GnRH2, and GnRH3 [2]. In the brains of vertebrates, GnRH1 is mainly present in the septo-hypothalamic area; chicken GnRH II (GnRH2) is universally present in the midbrain and evolutionarily conserved between taxa from fish to humans [3]. A third GnRH variant, GnRH3, is present in the terminal nerve ganglion of recently-derived teleosts [4]. It has been known for years that estrogen is one of the most critical factors affecting GnRH1 synthesis and secretion [5]. In fact, GnRH1 gene promoter contains functional estrogen response element (ERE) in mammals [6]. Furthermore, prenatal and adult GnRH1 neurons in rodents and GT1-7 GnRH-secreting neurons possess the ER subtypes (ERα and ERβ) mRNA and protein [7]. These results strengthen the assumption that estrogen may directly regulate gene expression in GnRH1 neurons. On the other hand, divergent results (see reviews [8,9,10]) and the failure to observe classical ERα protein and mRNA in GnRH1 neurons led to the general hypothesis that estrogen regulates GnRH1 neurons via nongenomic mechanisms such as transsynaptic [11], glial interactions [12], methylation [13], interaction with signaling kinases [14], or through membrane estrogen receptors [15].

In the past decade, kisspeptin (Kiss1) neurons have been demonstrated to be the most promising candidate as an indirect estrogenic modulator of GnRH neurons mainly in mammals because of (a) the expression of ER in kisspeptin neurons, (b) the estrogenic feedback action on kisspeptin neurons, and (c) the direct action of kisspeptin on GnRH neurons via kisspeptin receptor (Kiss1R = GPR54) [16]. Similar findings have been demonstrated in nonmammalian vertebrates including teleosts. Several teleost fish possess two genes encoding kisspeptin (Kiss1 and Kiss2) and their receptors (Kiss1R and Kiss2R) with different distribution and functions [17,18]. In medaka, hypothalamic Kiss1 but not Kiss2 neurons express ER and are sensitive to estrogen [19], while in goldfish, both Kiss1 and Kiss2 neurons express ER and are regulated by estrogen feedback [20]. Similarly, the expression of kisspeptin receptor types in GnRH neurons has been shown in some fish species, although there are still discrepancies among different fish species [21,22,23,24]. These findings indicate the possible involvement of kisspeptin neurons in estrogenic feedback regulation of GnRH neurons in fish. However, recent gene mutation studies in teleosts demonstrated a lack of significant impact of mutation of kisspeptin-GPR54 genes on the reproductive capability in fish [25], suggesting the existence of multiple mechanisms of GnRH regulation.

The genomic actions of estrogen are mediated through interactions of ER complexes with ERE on DNA. Apparently, the half-site of a consensus thyroid hormone response element is identical to ERE, and thyroid hormone receptors can bind to a consensus ERE [26]. In addition, multiple steroid hormone-binding elements have been identified on the GnRH promoter regions [27,28]. Thus, GnRH neurons could be influenced by various steroids in addition to estrogen. In addition to steroids, a series of xenobiotic chemicals have been identified as endocrine disrupting chemicals that are able to mimic and/or interfere with endogenous hormonal activity by binding to steroid nuclear receptors or xenobiotic nuclear receptors such as pregnane X-receptor (PXR) and c retinoid X receptors (RXR) [29]. Therefore, xenobiotic chemicals could affect GnRH neural functions through xenobiotic receptors. However, transcriptional regulation of GnRH genes via xenobiotic nuclear receptors has only been demonstrated in vitro in neuronal culture cells [30,31,32], but not in vivo in the brain.

To address this issue, we used an immunofluorescent-labeled cell harvesting procedure coupled with real-time PCR that allowed us to examine gene expression profiles in identified individual neurons [33]. Using the single-cell multiplex real-time PCR, we analyzed the gene expression of nuclear receptors for androgen (AR), estrogen (ER), glucocorticoid (GR), progesterone (PR), thyroid hormones (TR), and xenobiotics (RXR and PXR) in individual neurons of GnRH1, GnRH2, and GnRH3 in male cichlid fish tilapia *Oreochromis niloticus*. Further, we examined the expression of ER types (ERα, ERβ, and ERγ) in three GnRH types in male tilapia of different social statuses (dominant and subordinate).

## 2. Results

### 2.1. Plasma Hormone Levels

**Figure 1:**
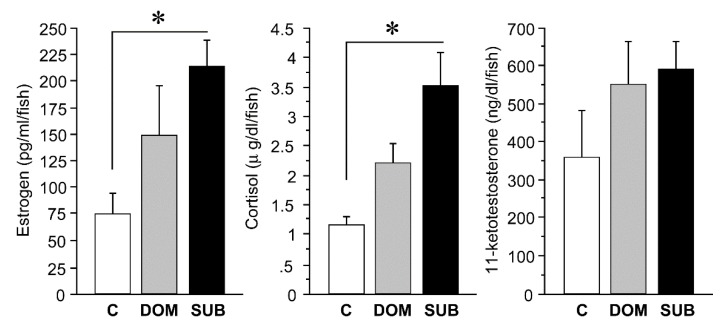
Histograms showing plasma hormone (estrogen, cortisol and 11-ketotestosterone) levels in control (C), dominant (DOM) and subordinate (SUB) males. * *p* < 0.05 significantly different when compared with control.

Plasma estrogen levels were significantly (*p* < 0.05) high in subordinate (211.9 ± 24.7 pg/mL) as compared with control (socially neutral males collected from home tank) males (72.4 ± 20.2 pg/mL), but there was no difference between dominant (146.8 ± 47.8 pg/mL) and subordinate or control males (Figure 1). Plasma cortisol levels were significantly (*p* < 0.05) high in subordinate (3.5 ± 0.6 µg/dL) as compared with control males (1.1 ± 0.1 µg/dL), but there was no difference between dominant (2.2 ± 0.3 µg/dL) and subordinate or control males (Figure 1). There was no difference in plasma 11-ketotestosterone (11-KT) levels among dominant (546.3 ± 114.7 ng/dL), subordinate (586.1 ± 72.2 ng/dL), and control males (352.1 ± 126.2 ng/dL) (Figure 1).

### 2.2. Expression of GnRH and Nuclear Receptor Genes in GnRH Neurons

**Figure 2:**
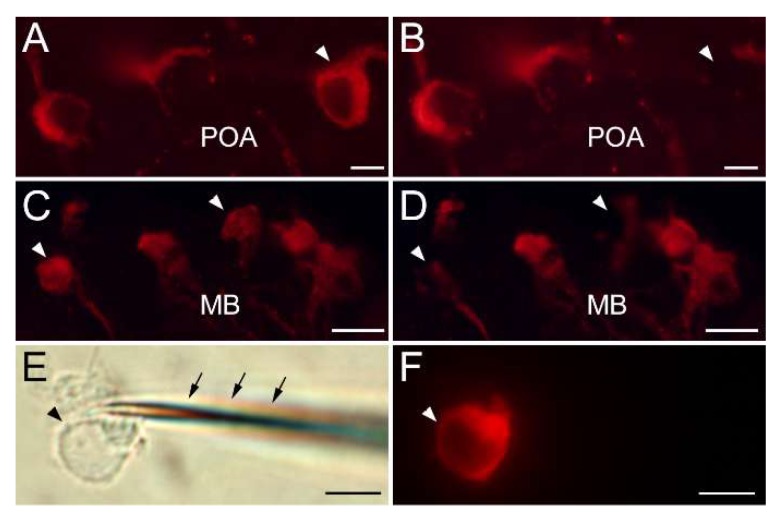
Photomicrographs of harvesting of Cy3-labeled immunofluorescent GnRH neurons. (**A**,**B**) GnRH1 neurons in the preoptic area (POA); (**C**,**D**) GnRH2 neurons in the midbrain (MB); (**E**,**F**) GnRH3 neurons in the terminal nerve. Arrows indicate the GnRH cells before (**A**,**C**) and after (**B**,**D**) harvesting. Scale bars = 20 μm.

Each antibody specifically labeled cells in only one of the three brain regions, i.e., in the preoptic area (GnRH1), midbrain tegmentum (GnRH2), and in the terminal nerve ganglion (GnRH3) (Figure 2). 

**Figure 3:**
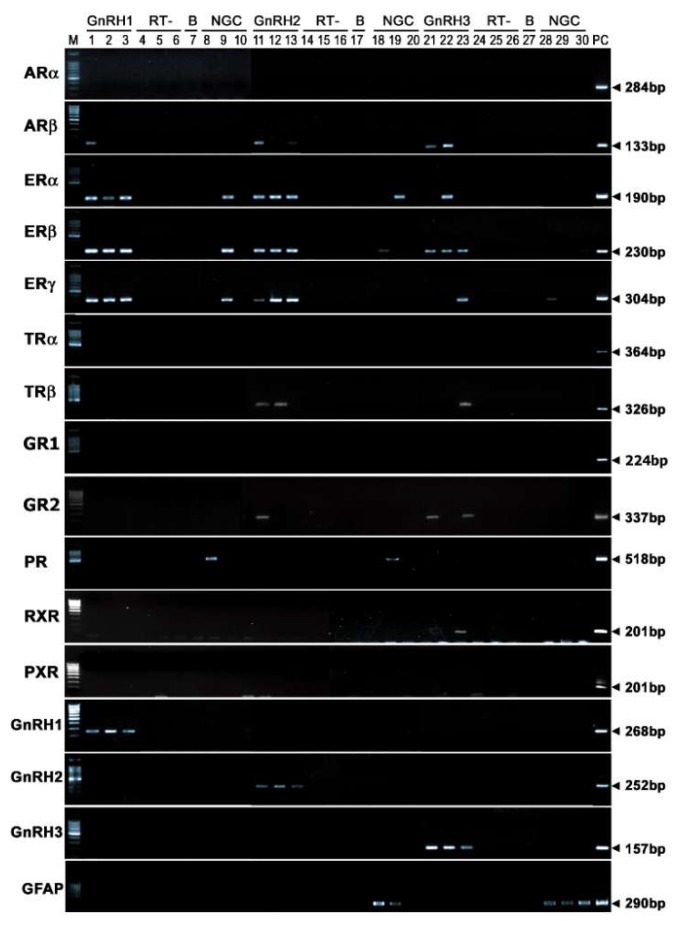
Composite gel showing GnRH and nuclear receptor mRNA expression in the three GnRH subtypes neurons taken from aggressive males (GnRH1, lane 1–3; GnRH2, lane 11–13; GnRH3, lane 21–23). Nested-PCR was necessary to observe ARβ. RT-, without reverse transcriptase (lanes 4–6, 14–16, 24–26). B, Buffer controls (lanes 7, 17, 27). NGC, Non-GnRH cells surrounding GnRH1-3 immunoreactive neurons (lanes 8–10, 18–20, 28–30). M, Marker, DNA 100 bp-size ladders (#N3231, for ARα, ERα, ERβ, ERγ, TRα, TRβ, GR1, PR, GnRH2, and GnRH3 or #i-Lad3 for ARβ, GR2, RXR, PXR, GnRH1, and GFAP). The sizes of the band pairs, is given in the right-hand margin.

There was no genomic DNA and glial cell contamination in the total RNA extracted from harvested single GnRH neurons (Figure 3). Using RT-PCR, 60–70% of GnRH1 and GnRH3 and 25% of GnRH2 neurons were seen to contain respective GnRH mRNA transcripts (Figure 3), whereas real-time PCR showed 80–90% of GnRH1, GnRH2, and GnRH3 neurons had GnRH mRNA transcripts. RT-PCR revealed the expression of transcripts for ARβ, ERα, ERβ, and ERγ in all three GnRH neuron types, while TRβ and GR2 expression was only detected in GnRH2 and GnRH3 neurons, and the expression of RXR mRNA was only detected in GnRH3 neurons (Figure 3). On the other hand, transcripts for ARα, GR1, PR, TRα, and PXR were undetectable in all GnRH neuron types. Among the nuclear receptors, estrogen receptors (ERα, ERβ, and ERγ) transcripts were further quantified by real-time PCR in GnRH neurons in male tilapia of two different social statuses. 

### 2.3. Quantitative Analysis of Estrogen Receptors in GnRH Neurons

**Figure 4:**
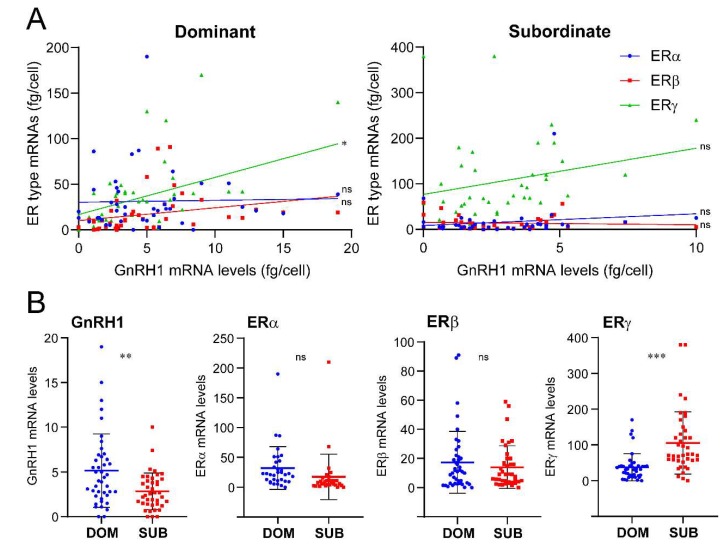
Expression of ER genes in GnRH1 neurons in dominant and subordinate male tilapia. (**A**) Graphs showing the expression of ERα (blue dots), ERβ (red dots), and ERγ (green dots) mRNA levels in individual cells in dominant and subordinate males. The X- and Y-axes represent the absolute mRNA levels (fg/cell) of GnRH1 and ER types in individual GnRH1 cells, respectively. (**B**) Scatter blot graphs showing mRNA levels of GnRH1 and ERα-γ in individual GnRH1 cells in dominant (DOM, blue dots) and subordinate (SUB, red dost) males. Correlation of GnRH1 and ER types mRNA levels were examined by Pearson’s r, while the mean difference of GnRH1 and ER types mRNA levels between DOM and SUB were analyzed by an unpaired t test. *p* values: ** *p* < 0.01, *** *p* < 0.001; ns, nonsignificant.

**Figure 5:**
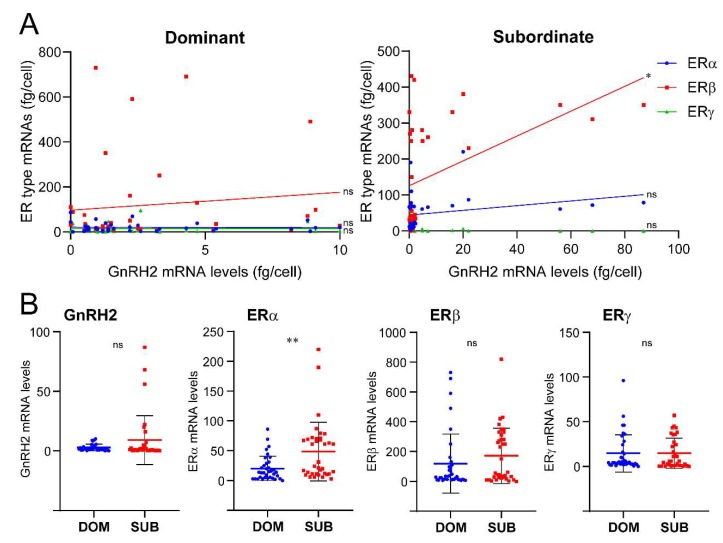
Expression of ER genes in GnRH2 neurons in dominant and subordinate male tilapia. (**A**) Graphs showing the expression of ERα (blue dots), ERβ (red dots) and ERγ (green dots) mRNA levels in individual cells in dominant and subordinate males. The X- and Y-axes represent the absolute mRNA levels (fg/cell) of GnRH2 and ER types in individual GnRH2 cells, respectively. (**B**) Scatter blot graphs showing mRNA levels of GnRH2 and ERα-γ in single GnRH2 cells in dominant (DOM, blue dots) and subordinate (SUB, red dost) males. Correlation of GnRH2 and ER types mRNA levels were examined by Pearson’s r, while the mean difference of GnRH2 and ER types mRNA levels between DOM and SUB were analyzed by an unpaired *t* test. *p* value: ** *p* < 0.01; ns, nonsignificant.

**Figure 6:**
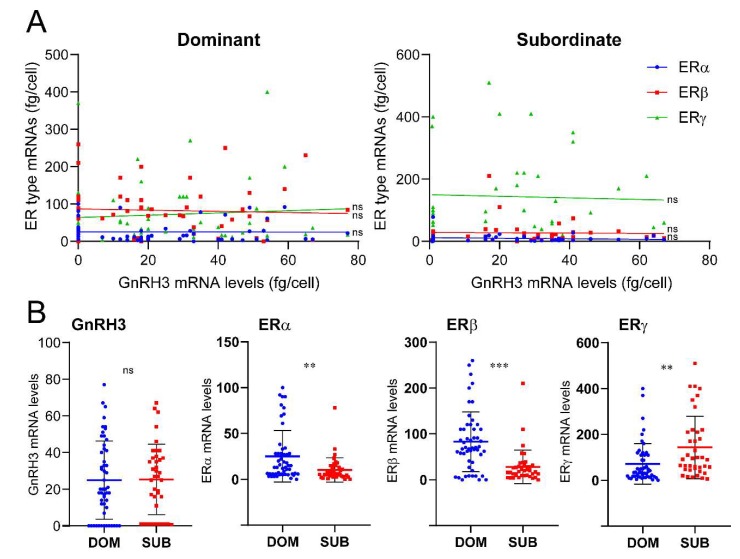
Expression of ER genes in GnRH3 neurons in dominant and subordinate male tilapia. (**A**) Graphs showing the expression of ERα (blue dots), ERβ (red dots) and ERγ (green dots) mRNA levels in individual cells in dominant and subordinate males. The X-and Y-axes represent the absolute mRNA levels (fg/cell) of GnRH3 and ER types in individual GnRH3 cells, respectively. (**B**) Scatter blot graphs showing mRNA levels of GnRH3, and ERα-γ in single GnRH3 cells in dominant (DOM, blue dots) and subordinate (SUB, red dost) males. Correlation of GnRH3 and ER types mRNA levels were examined by Pearson’s r, while the mean difference of GnRH3 and ER types mRNA levels between DOM and SUB were analyzed by an unpaired *t* test. *p* values: ** *p* < 0.01, *** *p* < 0.001; ns, nonsignificant.

Real-time PCR showed that all three GnRH populations expressed single or multiple ER types (Table 1). However, a large variation in mRNA levels of GnRH (GnRH1, GnRH2, and GnRH3) and ER types was observed between individual cells (Figure 4, Figure 5 and Figure 6). In addition, there were positive correlations between ERγ and GnRH1 (*p* > 0.01) in dominant male (Figure 4), and between ERβ and GnRH2 (*p* > 0.01) in subordinate males (Figure 5). 

GnRH1: Numbers of GnRH1 neurons expressing ER types were higher in dominant as compared subordinate males (Table 1). GnRH1 neurons co-expressing ERα and ERγ mRNAs were only seen in dominant males (22.0%). Expression levels of GnRH1 and ERγ mRNAs were positively correlated (*p* < 0.01) in dominant males (Figure 4A). GnRH1 mRNA levels were significantly higher in dominant as compared to subordinate males (*p* < 0.01), while ERγ mRNA levels in GnRH1 cells of subordinate males were significantly higher as compared to those in dominant males (*p* < 0.001) (Figure 4B). There was no significant difference in ERα and ERβ mRNA levels in GnRH1 cells between two social statuses.

GnRH2: Overall, there was no difference in the number of GnRH2 cells expressing ER types between the two social statuses, while a higher number of GnRH2 cells expressing only ERβ mRNAs were seen in subordinate males compared with those in dominant males (DOM 23.0% vs SUB 61.4%) (Table 1). On the other hand, a higher number of GnRH2 neurons coexpressing all three ER types were seen in dominant males compared to subordinate males (DOM 20.8% vs SUB 4.5%) (Table 1). Expression levels of GnRH2 and ERγ mRNAs were positively correlated (*p* < 0.01) in subordinate males (Figure 5A). There was no significant difference in GnRH2 mRNA levels between dominant and subordinate males (Figure 5B). GnRH2 and ERβ mRNA levels were positively correlated in subordinate males (*p* < 0.01), while ERα mRNA levels in GnRH2 cells were significantly higher in subordinate males compared to those in dominant males (*p* < 0.001) (Figure 4B). There was no significant difference in ERβ and ERγ mRNA levels in GnRH2 cells between the two social statuses.

GnRH3: There was no difference in the numbers of cells expressing ER types between the two social statuses. Higher numbers of GnRH3 cells coexpressing three ER types mRNAs were seen in dominant males compared with those in subordinate males (DOM 57.3% vs SUB 2.5%) (Table 1). On the other hand, GnRH3 cells expressing only ERβ mRNA were only seen in subordinate males (21.8%) (Table 1). There was no significant correlation between GnRH3 and ER types mRNA levels (Figure 6A). Collectively, there was no significant difference in GnRH3 mRNA levels between the two social statuses. Expression levels of ERα and ERβ mRNA in GnRH3 neurons were significantly (ERα, *p* < 0.01; ERβ, *p* < 0.001) higher in dominant males, while ERγ mRNA levels in GnRH3 neurons were significantly (*p* < 0.01) higher in subordinate males (Figure 6B). 

## 3. Discussion

### 3.1. Expression of Steroid and Xenobiotic Nuclear Receptors in GnRH Neurons

The localization of GnRH1 (preoptic area), GnRH2 (midbrain), and GnRH3 (terminal nerve) is consistent with our earlier observations using in situ hybridization and immunohistochemistry [21,33]. The control measures that we undertook demonstrate that our harvested cells were a pure population of GnRH neurons, because the amplicons were authentic, they did not arise from genomic DNA contamination, and there was no glial contamination because the absence of expression of the glial cell marker gene. However, possible contamination of other cell types or nonspecific cells cannot be ruled out because of possible nonspecific crossreactivity with other molecules. Using the single-cell gene profiling technique coupled with real-time PCR, we found that all GnRH1, GnRH2, and GnRH3 neuron types express ER genes. Similarly, the coexpression of ERα and ERβ in GnRH neurons has been demonstrated in mice using single-cell PCR [34]. Although immunohistochemical and *in situ* hybridization studies in mammals have previously shown the presence of ERα and ERβ in GnRH1 neurons [35,36,37], the presence of nuclear receptors including ER types in GnRH1 neurons has been debated over the last two decades because of contradictory results [12]. Primarily, the difficulties of detecting the expression of nuclear receptor genes or proteins in GnRH neurons are due to technical limitations owing to their low expression levels, i.e., below the detection level by *in situ* hybridization or immuohistochemistry [38,39]. The low levels or failure of detection of ER expression in GnRH neurons have been implicated in the untranslation of ER mRNA, or in the rapid degradation of ER protein [34,40]. 

The present study provides evidence of the coexpression of multiple nuclear receptor types (ERα, ERβ, ERγ, ARβ, GR2, and TRβ), including the xenobiotic receptors (PXR and RXR) in GnRH neurons. These findings correspond with the presence of response elements for ER, AR, GR, and TR in tilapia GnRH1, GnRH2, and GnRH3 gene promoters, and response elements for RXR in GnRH2 and GnRH3 gene promoters [27]. The presence of nuclear receptors in GnRH neurons suggests that GnRH neurons can be directly regulated by steroids and steroid-like chemicals in fish. This, however, does not rule out the possibility of other indirect steroid modulation of GnRH types. For example, a previous study on zebrafish demonstrated a close association between GnRH3 neurons and radial glial cells [41]. In some fish species, the expression of kisspeptin receptors (GPR54 types) in GnRH neurons, as well as the estrogenic modulation of kisspeptin neurons, have been demonstrated [42]. Therefore, sex steroids could modulate GnRH neurons via multiple action pathways, which, however, could vary, depending on species, developmental stage, and the physiological/gonadal status of animals. 

In several vertebrate species, glucocorticoids have been shown to alter GnRH1 neuronal activities [43,44,45]. However, in the present study, no expression of GR was observed in GnRH1 neurons in male tilapia. On the other hand, there was expression of GR mRNA in some GnRH2 and GnRH3 neurons. We have previously demonstrated the stimulatory effect of early-life exposure to the synthetic glucocorticoid, dexamethasone (DEX) on GnRH2 and GnRH3 mRNA levels in adult zebrafish [46]. Furthermore, the GnRH3 promoter appeared to be methylated in the DEX-treated fish. Therefore, glucocorticoid may act on GnRH2 and GnRH3 neurons via genomic and nongenomic mechanisms.

The expression of thyroid receptor (TRβ) in GnRH2 and GnRH3 neurons corresponds with our previous observation in male tilapia [47]. However, our previous study demonstrated the expression of TRβ in all GnRH neuron types, as well as the expression of TRα in GnRH1 and GnRH2 neurons, which were not observed in the present study. The expression of TR in preoptic GnRH neurons has also been demonstrated in sheep and hamsters [48]. This difference could be due to differences in the methodologies for sample preparation (single cell vs grouped cells) and physiological conditions of fish. In mammals, progesterone has been shown to directly act on GnRH neurons via PR [49,50]. However, no expression of PR mRNA was detected in GnRH neuron types in the male tilapia. In addition, no evidence for the direct action of progesterone on GnRH neurons has been reported in fish. However, in Atlantic croaker, 20β-S progestin (17,20β, 21-trihydroxy-4-pregnen-3-on) has been shown to negatively regulate GnRH1 secretion via its membrane receptor (mPR) in hypothalamic slice culture [51]. Therefore, in fish, progesterone may act on GnRH neurons through mPR. The presence of xenobiotic receptors in GnRH neurons suggests possible direct effects of xenobiotic chemicals, such as endocrine-disrupting chemicals (EDCs), on GnRH neurons. In fact, several EDCs that have been shown to alter GnRH systems, such as dichlorodiphenyldichloroethylene, phthalic acid, nonylphenol, and bisphenol A [52,53], and these EDCs could act on GnRH neurons via xenobiotic receptors. In addition, retinoic acid has been shown to influence the transcriptional regulation of the human GnRH2 gene expression [30], as well as aggressive behaviors in rats [54]. The presence of PXR mRNA expression was identified in high percentages of GnRH3 neurons in subordinate males. PXR is generally activated by xenobiotics, and induces cytochrome P450 3A4 (CYP3A4), resulting in altered drug metabolism and drug–drug interactions, in addition to enhanced metabolism of endogenous substrates including glucocorticoid [55]. Glucocorticoids play a critical role in CYP3A4 expression and induction through a process involving GR-mediated PXR mRNA accumulation [56]. Therefore, the coexpression of GR and PXR in GnRH3 neurons could be involved in the regulation of steroid biosynthesis by CYP3A4 in subordinate males. However, the physiological role of xenobiotic receptors in GnRH neurons and their physiological significance remain to be further evaluated. 

Importantly, teleost fish lack the hypophyseal-portal system called “median eminence”, and thus, GnRH fibers directly innervate to the pituitary, which may require an additional mechanism to drive the estrogen feedback to GnRH neurons. In terms of the molecular process, the classical genomic nuclear-initiated (ERE-dependent) effect of steroid feedback is much slower process than nongenomic membrane-initiated (or ERE-independent) effect. Therefore, the presence of nuclear receptors in GnRH neurons may be responsible for organizational effects within GnRH neurons, such as cell survival, and cellular or synaptic plasticity, rather than activational effects, such as the excitation of GnRH neurons or the stimulation of GnRH secretion. However, it is still unclear how such organizational effects of estrogen on GnRH neurons can be achieved independently in mammal ERα.

### 3.2. Functional Roles of Steroid and Xenobiotic Actions on GnRH Neurons and Social Status

A number of studies in mammalian and nonmammalian vertebrates have demonstrated the association between elevated androgen levels and social status [57]. However, in the present study, there was no difference in plasma androgen levels among dominant, subordinate, and intermediate males, as previously reported in other fish such as *Tilapia zillii*, *Neolamprologus pulcher,* and *Salvelinus fontinalis* [57]. It has been demonstrated in male tilapia that there is no correlation between androgen levels (both T and 11-KT) prior to group formation and the social status achieved, while strong correlations between androgen levels can be observed once fish acquired their social status [58]. Therefore, the male fish used in this study may not have fully established their social status, or their social status could have already been dissolved when they were captured. However, the high levels of plasma cortisol in subordinate males clearly indicate that these subordinate males are socially and chronically under stress. There was no difference in plasma estrogen levels between dominant and subordinate males as reported previously in another cichlid fish [59], while estrogen levels in subordinate males were significantly higher when they were compared with those in intermediate males. It is suggested that social experience has important effects on the production of estrogen within the brain [60]. The role of estrogen or its production (aromatization from testosterone) in socially aggressive/dominant animals is well documented [61], whereas, its possible role in stressed/subordinate animals has scarcely been reported. In female rats, both acute and chronic stress increase plasma estrogen levels [62,63]. Therefore, higher levels of estrogen in subordinate males could be associated with social stress rather than social status. 

Previously, a positive correlation between ER types and GnRH1 gene expression was demonstrated in the whole and olfactory region of the brain of another cichlid fish, *Astatotilapia burtoni* [64,65]. In mammals, it is apparent that ERs regulate the secretion and synthesis of GnRH in mature GnRH1 neurons [5,66]. In the present study, there was positive correlation between GnRH1 and ERγ mRNA levels in the GnRH1 neurons of dominant males. On the other hand, within single GnRH1 neurons, ERγ mRNA levels were higher in subordinate males as compared to those in dominant males. This may suggest that estrogen actively promotes GnRH1 synthesis (and maybe secretion) in dominant males, while estrogen may also act as a negative regulator of GnRH1 neurons under different social/physiological conditions. In fact, it has been shown that estrogen directly represses GnRH gene expression in GnRH-secreting GT1-7 hypothalamic cells [7]. However, it is also possible that the higher ERγ mRNA levels in GnRH1 neurons of subordinate males could be due to positive feedback of higher plasma estrogen levels. 

We also found that GnRH2 and ERβ mRNA levels were positively correlated in GnRH2 neurons of subordinate males, while at the single cell level, ERα mRNA levels were higher in subordinate males compared to dominant males. Although there was no correlation between GnRH3 and ER mRNA levels, at the single cell levels, ERα and ERβ mRNA levels were higher in dominant males, and ERγ mRNA levels were higher in subordinate males. Our previous study in the tilapia demonstrated the lack of effect of estradiol benzoate on GnRH2 and GnRH3 mRNA levels [67]. However, the differential expression levels of ER types in GnRH2 and GnRH3 neurons could indicate the presence of estrogenic feedback mechanism on nonphysiotropic GnRH types. In zebrafish, estrogen has been demonstrated to contribute to the regulation of neuronal development of GnRH3 neurons [68], although estrogen had no effect on the number of GnRH3 neurons in male tilapia [69]. However, there are still a couple of limitations to associating gene expression profiles within GnRH neurons with social status. For instance, the possible involvement of other parameters such as weight (general, or gonads), plasma hormonal levels, and the behavioral performance (aggression or stress) of individual animals on gene profiles in GnRH neurons, which could be associated with more profound changes of general physiology. The data would be more conclusive if a correlative analysis between weight and the aforementioned quantitative markers were included in future studies. Another potential limitation of the study is that gene expression profiles within GnRH1, 2, and 3 cells could have been influenced by the positions of each cell, as soma sizes or cell numbers of GnRH cells have been shown to be correlated with social or reproductive states in fish [70,71].

## 4. Materials and Methods

### 4.1. Animals

Fish were maintained in freshwater aquaria at 27 ± 1 °C with a controlled natural photo-regimen (14/10 h, light/dark cycle). The fish were maintained and used in accordance with the Guidelines of the Animal Ethics Committee of Monash University (Animal Ethics Approval Number, SOBSB/MY/2007/55, and Approved on 10 March 2008). 

Sexually-mature males of tilapia that continue to maintain territory in their home tank were size matched with two other males and placed together in a neutral experimental tank (60 L × 30 W × 45 H cm; 55 L). This cichlid fish species exhibits two classes of males: (i) dominant, aggressive, and reproductively-active, and (ii) socially-stressed, reproductively-inactive and subordinate [72]. Each tank had one clear aggressive male within the first 15 min after introduction. The socially-stressed males changed their body coloration to pitch dark and became motionless at the surface of the water. One hour after introduction, the aggressive male (standard length, SL: 11.6 ± 0.4 cm, body weight. BW: 52.6 ± 5.0 g, gonadosomatic index; GSI: 1.2 ± 0.2, *n* = 8) and the socially-stressed males (SL: 10.9 ± 0.2 cm, BW: 43.0 ± 2.2 g, GSI: 0.3 ± 0.1, *n* = 8) were anesthetized by immersion in a 0.01% solution of 3-aminobenzonic acid ethyl ester (MS222; Sigma, St. Louis, MO, USA) before they were killed by decapitation between 1400 and 1700 h. Blood samples were collected to determine levels of plasma steroid levels by radio-immunoassay (RIA). 

### 4.2. RIA of Plasma Estrogen, Testosterone and Cortisol Levels

After the behavior observation, blood samples were obtained from dominant (*n* = 8), subordinate (*n* = 8), and control (socially-intermediate and sexually-matured fish from the community tank, *n* = 5) males. About 100 µL blood was drawn from each fish into heparinized microsyringes from the caudal peduncle. The blood was stored at 4 °C for several hours and centrifuged at 4000 rpm to obtain the plasma. Plasma estrogen, testosterone, and cortisol were measured by RIA as previously described [73]. Although fish have two androgens (testosterone, T and 11-ketotestosterone, 11-KT), 11-KT was measured, as it is predominant in fish. The assay sensitivity was 50 pg/mL. The results were statistically analyzed by one-way ANOVA with Scheffe’s post-hoc tests.

### 4.3. Immunohistochemistry

Preparation of tissues and GnRH immunohistochemistry were carried out as described previously [33]. Briefly, the brains were dissected and fixed in RNase-free buffered 4% paraformaldehyde for 1 h, embedded in 5% gelatin, and then chilled on ice for 30 min. Using vibratome (Dousaka EM Co., Kyoto, Japan), 500 μm-thick coronal brain slices were made, starting from the rostral-most part of the olfactory bulb. The brain slices were incubated for 12 h with rabbit primary antisera against GnRH types anti-GnRH3 antiserum (# lot.2; Prof. K. Aida, the University of Tokyo, Tokyo, Japan; dilution of 1:3500) for brain slices 1000–2000 μm, anti-GnRH1 antiserum (# ISP-I; dilution of 1:4000) for brain slices 2000–3000 μm, and anti-GnRH2 antiserum (# ISP-II; dilution of 1:3500) for brain slices 4000–5000 μm from the rostral-most part of the olfactory bulb. Brain slices were then incubated at room temperature for 2 h with Cy3-conjugated antirabbit IgG (Jackson Immuno Research, West Grove, PA, USA; dilution of 1:400). 

The specificities of GnRH antibodies used in this study have been characterized previously. Lot.2 antibody is specific for GnRH3 (salmon GnRH, 100%) and has no crossreactivity to GnRH1 (seabream GnRH, <0.01%), but has a lower crossreactivity to GnRH2 (chicken GnRH-II, 1.58%) [74,75]. The specificities of immunoreactivity of the antisera have also been verified by localization in several teleost species including the Nile tilapia, Japanese flounder (*Paralichthys olivaceus*), and chub mackerel (*Scomber japonicus*), where lot.2 labeled GnRH neurons that are present in the olfactory bulb-terminal nerve (GnRH3), but not in the preoptic area (GnRH1) or midbrain tegmentum (GnRH2) [76,77,78]. Similarly, the antibodies to tilapia GnRH types (ISP-I and ISP-II) have been shown to specifically label cells in the preoptic area (ISP-I for GnRH1) and in the midbrain tegmentum (ISP-II for GnRH2), respectively [33]. These results are consistent with the localization of cells expressing GnRH type genes demonstrated by *in situ* hybridization [21,79].

### 4.4. Harvesting Immuno-Labelled GnRH Neurons

The harvesting of single immuno-labelled GnRH neurons was carried out as reported previously [33]. The brain slices were transferred into a culture dish containing lysis buffer (200 mM Tris-HCl, 200 mM NaCl, 1.5 mM MgCl_2_, 2% SDS, pH 7.5) and observed under a fluorescence microscope (Olympus BX50 WI, Olympus Optical Co. Ltd., Tokyo, Japan) fitted with a micromanipulator (Narishige, Tokyo, Japan). Micropipettes were fabricated from thin-walled borosilicate glass microcapillaries (1.5 mm outer diameter, Harvard Apparatus Ltd., Edenbridge, Kent), heated, and pulled using a micropipette puller (Type PE-2, Narishige, Tokyo). Immunofluorescent-labeled GnRH neurons were identified with a 10× water immersion objective lens and WIG filter (Olympus). With the help of the micromanipulator, the micropipette was brought into contact with the cell soma. Using a negative pressure, the cell was harvested into the micropipette under visual control and subsequently expelled into a sterile 1.5 mL reaction tube containing 50 μL of the lysis buffer and stored in −80 °C until the total RNA was extracted. Three different populations of immunolabeled single-GnRH neurons were harvested from the brain of aggressive (GnRH1 = 84; GnRH2 = 66; GnRH3 = 69 cells) and socially-stressed males (GnRH1 = 86; GnRH2 = 76; GnRH3 = 71 cells). For unbiased cell sampling, 8 to 12 morphologically well-identified cells were harvested at random (~6 cells per section) along the rostro-caudal extent of the whole population of each GnRH type in each animal (*n* = 8 animals/experimental group). Cells that were located individually were harvested, and only those that tested positive for each GnRH type but negative for glial fibrillary acidic protein (GFAP) by RT-PCR [33], and that were free from genomic contamination, were used for real-time PCR analysis (n = 8–12 cells/animal; 66–86 cells/GnRH type/experimental group).

### 4.5. Nuclear Receptors in GnRH Neurons

The conditions for RT-PCR were similar to those described previously [33]. Briefly, the harvested single-GnRH neurons were digested with 1 µg of proteinase K (Gentra Systems, Minneapolis, MN, USA) for 1 h at 53 °C. The cell lysate was incubated for 1 h at 37 °C with 1U RNase-free DNase I (Promega, Madison, WI, USA) to eliminate genomic DNA. Total RNA was extracted from the cell lysate using Mini RNA Isolation Kit (Zymo Research, Orange, CA, USA) and reverse transcribed to cDNA with 1 pmol of oligo(dT)_12–18_ primer (Life Technologies, Inc., Carlsbad, California, USA) using 0.1 µL of SensiScript reverse transcriptase (Qiagen, Hilden, Germany).

To confirm the presence of nuclear receptor mRNA transcripts, we cloned and identified partial sequences of PR, RXR, and PXR in the tilapia (DDBJ/EMBL/GenBank accession nos.: PR, AB110982; RXR, AB111359; PXR, AB498797), whereas the sequences of other tilapia nuclear receptors were obtained from the DDBJ/EMBL/GenBank database (accession nos.: ARα, AB045211; ARβ, AB045212; ERα, U75604; ERβ, AB110981; ERγ, U75605; GR1, AB245405; GR2b, AB245406; TRα1, AF302248; TRα2, AF302249; TRβ, AF302247). Sequences of all primers are listed in Table S1. One tenth of a single-cell’s cDNA solution was subjected to RT-PCR using gene-specific primers for GnRH types and nuclear receptors (Table S1). As a positive control, 100 ng of whole brain cDNA was subjected to PCR. Buffers without harvested cells, no-RT, and non-GnRH cells were also subjected to PCR as negative controls. For GnRH and non-GnRH cells, GFAP primers (Table S1) were also included in the PCR protocol. The DDBJ/EMBL/GenBank accession numbers of the PCR primers for three GnRH types and GFAP in tilapia are as follows; GnRH1, AB101665; GnRH2, AB101666; GnRH3, AB101667, and GFAP, AB109167. The amplification products were resolved on 2% agarose gel with 100 bp molecular weight DNA ladders (#N3231, New England BioLabs, Beverly, MA, USA or #i-Lad3, i-DNA Biotechnology, Kuala Lumpur, Malaysia). All PCR products were further confirmed by sequencing.

### 4.6. Real-Time PCR for GnRH, ER, and Xenobiotic Receptors in GnRH Neurons

Quantitative analysis was performed for three GnRH types (GnRH1, GnRH2, and GnRH3) and ER types (ERα, ERβ and ERγ) using real-time PCR. cDNA samples from single-cells were subjected to real-time PCR, which was performed in duplicate in 10 µL reaction volumes consisting of 1× TaqMan Universal PCR Master Mix (Applied Biosystems, Foster City, CA, USA), 300 nM of each forward and reverse primers, 200 nM of TaqMan probe for GnRH types and ER types (Table S1), and one tenth of a single cell’s cDNA or control plasmid DNA containing target gene cDNA fragments using an ABI PRISM 7700 Sequence Detection System (Applied Biosystems). Amplification was performed at 50 °C for 2 min, 95 °C for 10 min, 45 cycles of 95° C for 15 s, and 60 °C for 1 min. The absolute amounts of transcripts were determined by establishing a linear amplification curve from serial dilutions (0.01 fg to 100 fg) of a plasmid DNA containing a GnRH or a nuclear receptor cDNA fragment. For each animal, the average mRNA levels of GnRH type and nuclear receptors per cell were determined, and these values were combined to give experimental group means. All values are presented as means (±SEM). Correlations of GnRH and ER types mRNA levels were examined using the Bravais-Pearson correlation test, while the mean difference of GnRH and ER types mRNA levels between DOM and SUB were analyzed using an unpaired Student’s t test or nonparametric ANOVA, followed by a post hoc Dunn’s multiple comparison test. The Tukey’s criterion applied to each group of data sets, allowing us to detect outliers [80]. *p* < 0.05 was considered statistically significant.

## 5. Conclusions

In the present study, we provided evidence for a direct steroid feedback action on three GnRH neuron types in fish. Differential expression patterns and levels of ER types in GnRH neuron types under different socio-reproductive statuses indicated the functional involvement of estrogenic actions on GnRH physiology. Using a single-cell gene expression assay, we revealed the presence of variations in nuclear receptor expression (and possibly steroid sensitivities) among individual GnRH cells, which may be influenced by physiological conditions such as social/reproductive status. More diversified analyses of molecular factors and the involved pathways within GnRH neurons need to be carried out to verify their physiological impacts on GnRH functions. 

## Figures and Tables

**Table 1:** Percentages of GnRH neuron types with single or multiple nuclear receptor mRNA transcripts in dominant (DOM) and subordinate (SUB) males (*n* = 8 each).

NR Types	GnRH1		GnRH2		GnRH3	
	DOM	SUB	DOM	SUB	DOM	SUB
ERα	16.1 ± 2.6	14.2 ± 4.8	5.0 ± 5.0	5.0 ± 5.0	4.0 ± 4.0	7.5 ± 4.8
ERβ	7.0 ± 4.4	16.3 ± 3.8	23.0 ± 7.8	61.4 ± 11.4	0.0	21.8 ± 4.5
ERγ	2.3 ± 2.3	7.1 ± 4.7	5.0 ± 5.0	9.5 ± 0.5	5.8 ± 2.4	17.0 ± 2.4
ERα+β	11.9 ± 4.9	9.6 ± 4.1	17.9 ± 6.9	9.5 ± 0.5	3.8 ± 2.3	15.0 ± 5.0
ERβ+γ	7.0 ± 4.4	9.2 ± 3.4	5.0 ± 5.0	5.0 ± 5.0	11.9 ± 3.7	19.1 ± 7.4
ERα+γ	22.0 ± 4.6	0.0	14.2 ± 6.3	0.0	13.9 ± 2.8	2.3 ± 2.3
ERα+β+γ	27.2 ± 5.1	12.1 ± 4.9	20.8 ± 7.5	4.5 ± 4.5	57.3 ± 7.0	2.5 ± 2.5
ER positive	91.0 ± 3.7	68.3 ± 6.9	90.9 ± 9.1	95.0 ± 5.0	96.8 ± 2.0	85.2 ± 6.5
ER negative	9.0 ± 3.7	31.7 ± 6.9	9.1 ± 9.1	5.0 ± 5.0	3.2 ± 2.0	14.8 ± 6.5

## Supplementary Materials

Supplementary materials can be found at https://www.mdpi.com/1422-0067/21/8/2724/s1.
