# Supplementary material for: Single-Cell Gene Profiling Reveals Social Status-Dependent Modulation of Nuclear Hormone Receptors in GnRH Neurons in a Male Cichlid Fish
Source: Int J Mol Sci. 2020 Apr 15;21(8):2724. doi: 10.3390/ijms21082724 (PMC7215790; doi:10.3390/ijms21082724)
Supplement: Supplementary file 1 [file ijms-21-02724-s001.pdf]

Supplementary Table S1: Sequence of PCR primers and fluorogenic probes used for real-time PCR.

RT-PCR.	Sequence.	Real-time PCR.	Sequence..
GnRH1-F.	5′-GGGATCTGGACAACTTCTCA-3′ .	Q-GnRH1-F.	5′-CTCGCAGGGACGGTGTTT-3′.
GnRH1-R.	5′-TTCTTGAATGTCCGGTGTC-3′ .	Q-GnRH1-R.	5′-TCTTCCCTCCTGGGCTCAGT-3′.
GnRH2-F.	5′-GAGCTGGACTCCTTTGGGAC-3′ .	Q-GnRH1	5′-CACAGGGCTGCTGTCAACACTGGTCATA-3′.
GnRH2-R.	5′-ACAAAATCACGTCAAGGCAG-3′ .	Probe.	5′-TGGTCCCATGGTTGGTATCC-3′.
GnRH3-F.	5′-AAGAGAAGTGTGGGAGAGCT-3′ .	Q-GnRH2-F.	5′-CCCTGCTTCACACAGCTTAATCT-3′.
GnRH3-R	5′-GTGCTGCTAATAATGATGTA-3′.	Q-GnRH2-R.	5′-AAATCTCTGATGTCCCAAAGGAGTCCAGCT-3′.
.	5′-GATCTCCCACCGGAAATGT-3′.	Q-GnRH2	5′-TGCTGGCGTTGGTGGTT-3′.
ARα F.	5′-GCGGCCCTCTTGAAGAATAC-3′.	Probe.	5′-CCTCAAGCTCTCCCACACTTCT-3′.
ARα R.	5′-TCCTTCCCTGAATTCCCTTT-3′.	Q-GnRH3-F.	5′-CAGCACTGGTCCTATGGATGGCTACC-3′ .
ARβ F.	5′-GCACTCAGCGCTTAACTCCT-3′.	Q-GnRH3-R.	5′-GCCTGCGACAGAGACAAACC-3′ .
ARβ R.	5′-GTAAGGACCGTGGACGTGTT-3′.	Q-GnRH3	5′-CACGCTTCCTTCCATCCTGA-3′.
ERα F.	5′-GACGAGAGGACAGGATTGGA-3′.	Probe.	5′-CCTGCCACACACAAGGGCGTCC-3′.
ERα R.	5′-GGCTCCAGTAAAGCCACAGA-3′.	Q-ERα F.	5′-AAGCCACAGAGAGGGTGAGC-3′.
ERβ F.	5′-AGAGACGATGGGCTGTCAGT-3′.	Q-ERα R.	5′-TATCCCCACAGGAGGGCTG-3′.
ERβ R.	5′-TGGCAGGACTGAGAAGGAGT-3′.	Q-ERα	5′-CCCCGGGACTTCTGCCTGCTATG-3′.
ERγ F.	5′-TTGTTTCATGGGACAGACCA-3′.	Probe.	5′-GCTATGACGGACGCTCTGG-3′.
ERγ R.	5′-GCCTGATTGAGGAGAACAGG-3′.	Q-ERβ F.	5′-CGAGTGTACTGCTGCCGGA-3′.
TRα F.	5′-CAGCCCTTCAACAGGATGAT-3′.	Q-ERβ R.	5′-CCATCGGCAAAACCGGCCTCA-3′.
TRα R.	5′-GTTGCATGGAGATCATGTCG-3′.	Q-ERβ Probe.	..
TRβ F.	5′-ATGTGCCACTTTGTGTTTGC-3′.	Q-ERγ F.	
TRβ R.	5′-CATTGTGAAGCGTGAGGAGA-3′.	Q-ERγ R.	
GR1 F.	5′-GGAAGAGCAGAGGTTTGACG-3′.	Q-ERγ Probe.	
GR1 R.	5′-CCCTACATGACCGACCAGTT-3′.	.	
GR2 F.	5′-ACTCCACGCTCAGGGATTTA-3′.		
GR2 R.	5′-AGTTTACACATCAATGACCA-3′ .		
PR F.	5′-ATGACTTCTGACATCATCTC-3′.		
PR R.	5′-CCAGTGCAGTCAACGAGGAG-3′.		
RXR-F1.	5′-GACAAGCAGCTCTTCACCCT-3′.		
RXR-F2.	5′-ACTGTGGGCACTGTTCCTGT-3′.		
RXR-R.	5′-CCATTGGCATGTGCAAAGA-3′.		
PXR-F1.	5′-TAACACCCCAGTTCAGCTTTC-3′.		
PXR-F2.	5′-CAGGCGTTTTTAGGGCACT-3′.		
PXR-R.	5′-TGGTATCGCTCGAAGTTTGC-3′ .		
GFAP F.	5′-AGGTCCTGGTACTCCTGCA-3′.		
GFAP R.			

Foot note: All probes were labeled with FAM dye for a 5′-reporter dye and TAMRA dye for 3′-quencher dye.
